# The role of mitochondrial haplogroups in glaucoma: a study in an Arab population

**Published:** 2008-03-13

**Authors:** Khaled K. Abu-Amero, Jose Morales, Thomas M. Bosley, Gamal H. Mohamed, Vicente M. Cabrera

**Affiliations:** 1 Mitochondrial Research Laboratory, Department of Genetics, King Faisal Specialist Hospital, Riyadh, Saudi Arabia; 2 Glaucoma Division, King Khaled Eye Specialist Hospital, Riyadh, Saudi Arabia; 3 Neuro-ophthalmology Division, King Khaled Eye Specialist Hospital, Riyadh, Saudi Arabia; 4 Department of Biostatistics, Epidemiology and Scientific Computing, King Faisal Specialist Hospital, Riyadh, Saudi Arabia; 5 Department of Genetics, Faculty of Biology, University of La Laguna, Tenerife, Spain

## Abstract

**Purpose:**

Glaucoma prevalence can vary geographically and ethnically, which suggests that a genetic element could play a significant role. Studies investigating the role of various mitochondrial haplogroups in the pathogenesis of glaucoma are scarce.

**Methods:**

We compared the prevalence of different mitochondrial haplogroups in 107 glaucoma patients (49 primary open-angle glaucoma, POAG; 29 primary angle-closure glaucoma, PACG; and 29 pseudoexfoliation glaucoma, PEG) and 552 maternally unrelated normal controls. All patients and controls were Saudi Arabs.

**Results:**

There was no statistically significant difference between patients and controls for all mitochondrial haplogroups tested except for PACG patients with mitochondrial haplogroup preHV1 (odds ratio=4.9; 95% CI 2.3 – 10.5; p=0.00002).

**Conclusions:**

Patients with preHV1 mitochondrial haplogroups are at higher risk of developing PACG. However, our study group is relatively small and further studies with more patients in other populations are needed to confirm this interesting finding.

## Introduction

During evolution, several mutations have accumulated in mitochondrial DNA (mtDNA), representing specific single nucleotide polymorphisms (SNPs), allowing human populations to be categorized into various mtDNA haplogroups. In certain populations, these haplogroups were found to confer resistance against type 2 diabetes [1], influence energy dependent processes such as sperm motility and the risk of developing late onset neurodegenerative diseases [2], and contribute to the development of various types of cancer [3-7], Parkinson disease [8], and multiple sclerosis [9].

Glaucoma prevalence can vary by geography and by ethnicity [10-13], suggesting that a genetic element could play a significant role. In the western world, in predominantly Caucasian groups for instance, primary open-angle glaucoma (POAG) is the most commonly encountered adult onset type of glaucoma [14] while primary angle-closure glaucoma (PACG) has been reported much more frequently in Asiatic populations [15-17]. Pseudoexfoliation glaucoma (PEG) has also demonstrated wide prevalence variation around the world [13].

Studies investigating the role of various mitochondrial haplogroups in the pathogenesis of glaucoma are scarce. Searching the literature, we came across only one study, which studied the role of mitochondrial haplogroups in POAG. Their haplogroup analysis was based on the phylogenetic network for European mtDNA [18], and the results of this study concluded that mitochondrial haplogroups H, T, J, U, K, W, I, V, X, and M do not appear to contribute to the pathogenesis of POAG [19]. On the other hand, there is quite a fair number of studies investigating the role of mitochondrial haplogroups in the pathogenesis of Leber hereditary optic neuropathy (LHON). LHON is another optic nerve disease which shares some similarities with certain types of glaucoma. LHON is mainly caused by one of three mitochondrial DNA mutations (11778 G>A, 14484 T>C, and 3460 G>A), which are known as the primary LHON mutations. There is a well established strong association between the mtDNA genetics background and both the 11778 G>A and the 14484 T>C LHON mutations but not the 3460 G>A. Meta-analysis of the available data has shown that individuals with the 14484 T>C mutation are 27 times more likely to belong to western Eurasian haplogroup J than control subjects and that individuals with the 11778 G>A mutation are three times more likely to belong to haplogroup J than control subjects. This was observed in western Eurasian populations [20]. The reason for this association is not clear, but one likely theory is that functional variants in complex I mitochondrial gene interacting synergistically with the primary LHON mutation are leading to further compromise of complex I function [21]. Here, we investigated the possible association of mitochondrial haplogroups with the pathogenesis of the three most common types of glaucoma (POAG, PACG, and PEG) prevalent in the Saudi Arab population.

**Figure 1:**
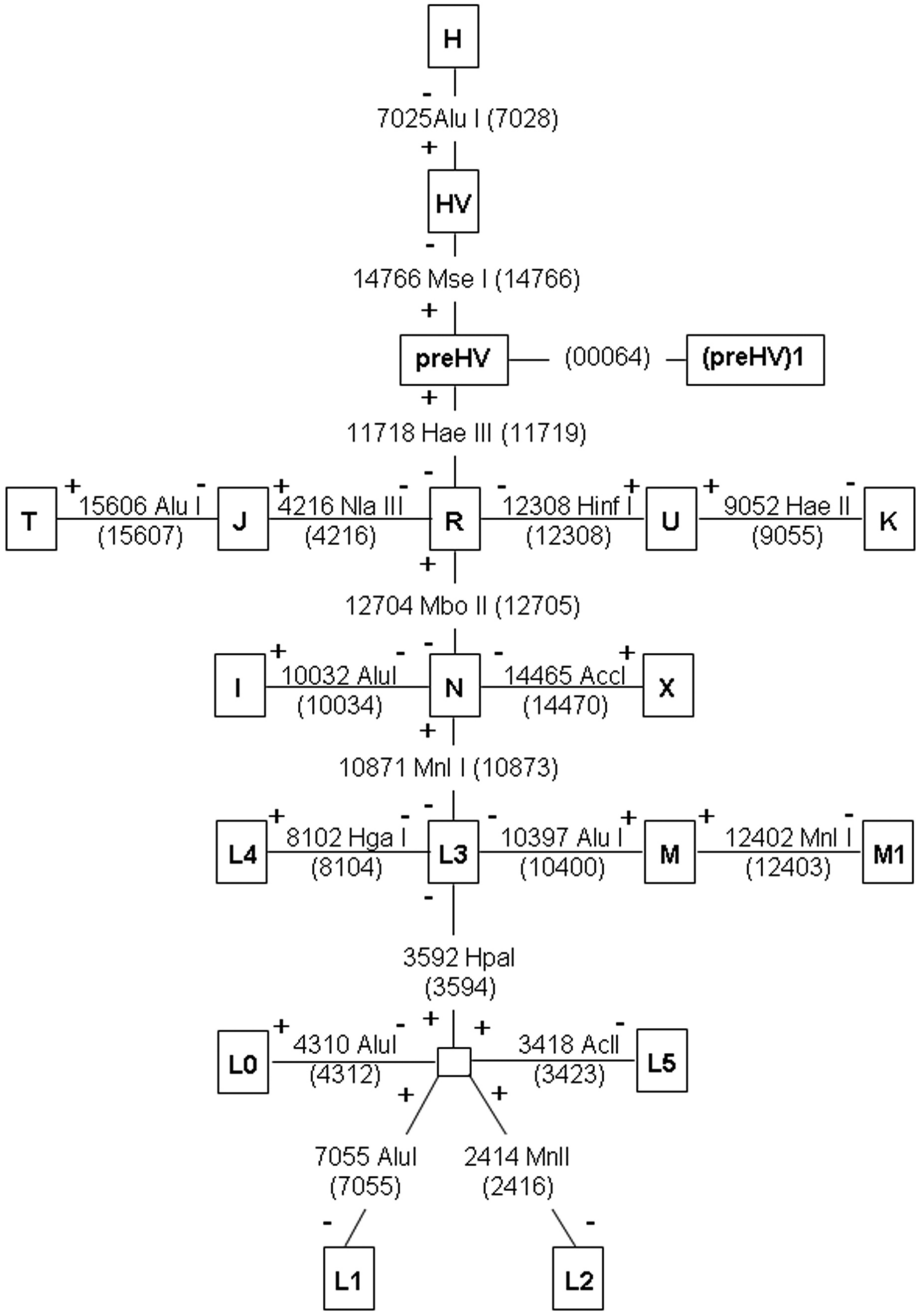
A schematic representation of diagnostic RFLPs (or variable positions sequenced) used to assort mtDNA haplotypes. Phylogenetic relationships of all the haplogroups detected in the glaucoma cohort studied are graphically represented. Diagnostic positions, detected by RFLP or sequencing, are depicted on the branches relating haplogroups. Positive signs (+) indicate restriction-site gains and negative signs (-) indicate restriction-site losses.

## Methods

### Patient enrollment

A total of 107 glaucoma patients (49 POAG, 29 PACG, and 29 PEG) were included in this study. The inclusion and exclusion criteria for each type of glaucoma were detailed elsewhere [22-24]. All patients were Saudi Arabs. Patients were selected from the Glaucoma Clinic at King Khaled Eye Specialist Hospital (KKESH) after examination by a glaucoma specialist (J.M.) and informed consent approved by the KKESH-IRB. Records were reviewed, and full ophthalmologic examinations were performed. Patients had either Goldmann manual kinetic perimetry (Haag Streit International, Koeniz-Bern, Switzerland) or Humphrey automated white on white stimulus static perimetry (Humphrey Field Analyzer II, Humphrey Systems, Dublin, CA) or both. Optical Coherence Tomography was performed with the OCT3 Unit by Humphrey Systems (San Leandro, CA) on some patients. Fundus photos were obtained using a Zeiss FF 450 system and conventional film. This research followed the tenets of the Declaration of Helsinki. Family members were not evaluated clinically or genetically.

**Table 1:** Haplogroup distribution in glaucoma patients and controls.

**Mitochondrial haplogroup**	**Controls** **(n=552)**	**Glaucoma patients (n=107)**	**Odds ratio**	**95% C.I.**	**p-Value**
H	47 (8.5%)	7 (6.5%)	0.75	0.30–1.79	0.62
I	5 (0.9%)	1 (0.9%)	1.03	0.17–6.20	1
J	116 (21%)	22 (20.6%)	0.97	0.56–1.67	0.98
K	22 (4%)	4 (3.7%)	0.94	0.27–2.95	1
L0	6 (1.1%)	1 (0.9%)	0.86	0.14–5.44	1
L1	3 (0.5%)	2 (1.9%)	3.49	0.40–25.9	0.19
L2	20 (3.6%)	6 (5.6%)	1.58	0.555–4.29	0.41
L3	22 (4%)	5 (4.7%)	1.18	0.38–3.39	0.79
L4	1 (0.2%)	2 (1.9%)	10.5	0.74–294.9	0.07
L5	4 (0.7%)	2 (1.9%)	2.61	0.33–16.70	0.25
M	17 (3.1%)	0	0.15	0.009–2.46	0.09
M1	19 (3.5%)	6 (5.6%)	1.67	0.58–4.56	0.27
N	41 (7.4%)	1 (0.9%)	0.12	0.01–0.81	0.02
preHV1	99 (17.9%)	31 (29%)	1.87	1.13–3.06	0.0127
R	17 (3.1%)	0	0.15	0.009–2.46	0.09
T	34 (6.2%)	4 (3.7%)	0.59	0.17–1.80	0.45
U	58 (10.5%)	10 (9.4%)	0.88	0.41–1.85	0.85
W	6 (1.1%)	0	0.425	0.024–7.67	0.59
X	15 (2.7%)	3 (2.8%)	1.03	0.23–3.88	1.0

Since we have 19 mitochondrial haplogroups, the Bonferroni correction should be 0.05/19=0.0026. Thus, a p-value<0.0026 was considered significant.

### Control enrollment

Control subjects were blood donors at the King Faisal Specialist Hospital and Research Centre who represented the spectrum of Saudi Arabs. Buccal swabs or peripheral blood were obtained from 552 maternally unrelated Saudi Arabs, all whose known ancestors were of Saudi Arabian origin. All control subjects reported no symptomatic, metabolic, genetic, or ocular disorders on an extensive questionnaire about family history, past medical problems, and current health.

### RFLP (sequencing) analyses of haplogroup diagnostic positions

To detect coding-region diagnostic haplogroup polymorphisms, a fragment spanning the diagnostic position was amplified using any of the 32 overlapping pairs of primers that cover the whole mtDNA genome, and the PCR conditions previously published [25]. However, a polymorphism at nucleotide position 12,308 was amplified using a reverse mismatch primer as described by Torroni et al. [26]. Amplified fragments were digested with the appropriate restriction endonuclease according to the supplier’s recommendations. Alternatively, the amplified fragments were analyzed by sequencing. For Eurasian haplogroups (H, HV, preHV, J, T, R, U, K, I, N, X and M) diagnostic positions were recompiled from Richards et al. [27]. For African haplogroups, L0, L1, and L3 from Chen et al. [28] and for L2, L4, and L5 from Kivisil et al. [29]. Finally, diagnostic positions for (preHV)1 were taken from Abu-Amero et al. [30] and for M1 from Gonzalez et al. [31].. Figure 1 shows a schematic representation of diagnostic RFLPs (or variable positions sequenced) used to assort mtDNA haplotypes.

### Data analysis

The frequency of each haplogroup among cases and controls were compared with the Χ^2^ test (Fisher’s exact test where appropriate), and the risk of having the disease if you have a certain haplogroup as compared to not having that specific haplogroup was estimated by computing odds ratio and its confidence interval. A p-value less than 0.05 was considered significant. Bonferroni correction was used to adjust the significance level of a statistical test to protect against Type I errors when multiple comparisons were being made. Since we have 19 mitochondrial haplogroups, the Bonferroni correction should be 0.05/19=0.0026. Therefore, a p-value less than 0.0026 was considered significant. In the case of comparing the haplogroup distribution among each of the three different glaucoma groups with the controls, the threshold was further reduced to 0.00087. All analyses were performed using SPSS v.13 statistical analysis software (SPSS Inc., Chicago, IL).

## Results

Our cohort consisted of 107 glaucoma patients (49 POAG, 29 PACG, and 29 PEG) and 552 ethnically matched healthy controls. Table 1 shows the mitochondrial haplogroup distribution among the glaucoma patients as one group and the controls as another group. There was no statistically significant difference between patients and controls for all mitochondrial haplogroups tested. We then compared the mitochondrial haplogroup distribution for each type of glaucoma separately with the controls (Table 2). There was no statistically significant difference between patients and controls for all haplogroups tested except among PACG patients with haplogroup preHV1 (odds ratio=4.9; 95% CI 2.3 – 10.5; p=0.00002).

**Table 2:** Haplogroup distribution in patients with various types of glaucoma and controls.

**Mitochondrial haplogroup**	**Controls (n=552)**	**Glaucoma patients (n=107)**					
		**PACG (n=29)**	**p-Value**	**PEG (n=29)**	**p-Value**	**POAG (n=49)**	**p-Value**
H	47	1	0.49	1	0.49	5	0.6
I	5	0	1	0	1	1	0.4
J	116	3	0.24	10	0.1	9	0.85
K	22	2	0.34	2	0.34	0	0.24
L0	6	0	1	0	1	1	0.45
L1	3	0	1	0	1	2	0.06
L2	20	5	0.005	0	0.62	1	1
L3	22	0	0.62	0	0.62	5	0.06
L4	1	0	1	0	1	2	0.02
L5	4	0	1	0	1	2	0.08
M	17	0	1	0	1	0	0.35
M1	19	1	1	1	1	4	0.11
N	41	0	0.25	0	0.25	1	0.24
preHV1	99	15	**0.00002**	8	0.22	8	1
R	17	0	1	0	1	0	0.38
T	34	1	1	2	0.69	1	0.35
U	58	0	0.1	4	0.54	6	0.63
W	6	0	1	0	1	0	1
X	15	1	0.56	1	0.56	1	1

Since we have 19 mitochondrial haplogroups, the Bonferroni correction should be 0.05/19=0.0026. Therefore, a p-value<0.0026 was considered significant. In the case of comparing the haplogroup distribution among each of the three different glaucoma groups with the controls, the threshold was further reduced to 0.00087. The only significant p-value is in bold.

## Discussion

Glaucoma is the most common optic neuropathic process affecting humans and the second most common cause of blindness worldwide [32]. Previous studies have demonstrated that glaucoma affects certain ethnic groups disproportionately (see Introduction). The prevalence of different types of glaucoma in Saudi Arabia is largely unknown. We found one eye population survey where glaucoma specialists were involved in the screening of 565 individuals older than 60. This study found that the prevalence of PACG was as frequent as POAG and approaching that of one reported in some Asian populations [33].

By studying 107 glaucoma patients of Saudi descent, we demonstrated that certain mtDNA haplogroups influence the development of certain types of glaucoma. We found that individuals with preHV1 haplogroup were at higher risk of developing PACG (p<0.00002). Although this association is statistically significant, it has to be stated that the PACG group analyzed here were small (n=29) and preHV1 is the second most common mitochondrial haplogroup among the Saudi population (17.9%) [34], which may raise some concern regarding this finding. Also, as the genetic diversity in Saudi Arabia is smaller than in other Middle East areas [30,34], this association could be even less significant. Additional association studies with more patients may be needed to eliminate casual susceptibility to PACG among individuals with preHV1 haplogroup.

We demonstrated an association between a specific mitochondrial haplogroup and a certain type of glaucoma in the Saudi Arab population. However, our study group is relatively small and from a relatively homogenous population. Thus, further studies with more patients and in various populations are needed to confirm our finding. We also cannot ignore the fact that genetics or environmental factors other than mitochondrial haplogroups could account for the occurrence of glaucoma.
